# Primary systemic amyloidosis initially presenting with digestive symptoms: a case report and review of the literature

**DOI:** 10.1186/s13000-015-0407-9

**Published:** 2015-09-21

**Authors:** Xiu Lin, Yueping Mao, Qing Qi, Chuyi Zhang, Yongzhen Tian, Yanyang Chen

**Affiliations:** Department of Dermatology, Second Affiliated Hospital, Sun Yat-Sen University, Guangzhou, 510120 China; Department of Dermatology, First Affiliated Hospital, Guangzhou University of Traditional Chinese Medicine, Guangzhou, 510405 China; Department of Pathology, First Affiliated Hospital, Sun Yat-Sen University, Guangzhou, 510120 China

**Keywords:** Primary systemic amyloidosis, Gastrointestinal symptoms, Biopsy, Congo Red staining, Plasmacytosis, M proteinemia

## Abstract

Primary systemic amyloidosis (PSA) is one of systemic amyloidosis, characterized by clonal plasma cell disorder. The disease is rare and with high fatality. Signs and symptoms of PSA are various and complex, which depend on the organs involved. Here we report a case in which the patient initially suffered from gastrointestinal symptoms. Gradually periorbital purpura, skin fragility, and subsequent petechiae, ecchymoses and sclerosis of the distal limbs, appeared. Biopsy of his palmar skin showed scleroderma-like changes. However, histopathology of the petechiae lesion on forehead with Crystal Violet Staining prompted deposition of amyloid; gastric mucosal biopsy with Congo Red staining was also positive, which made clear the diagnosis of PSA. Bone marrow biopsy and serum immunofixation electrophoresis (IFE) revealed plasmacytosis and M proteinemia. Other examinations were performed to assess the function of organs. PSA was challenging due to the initial atypical clinical presentation and absence of biopsy with special staining. The case demonstrates that PSA should be considered in patients with multisystemic symptoms and biopsy with Congo Red staining should be performed to exclusively diagnose amyloidosis.

## Background

Primary systemic amyloidosis (PSA), also called Lubarsch-Pick disease, is a rare, complex and protean disease. Currently, it is a mortal disease without any effective therapy, and its median survival time is approximatedly 13 months [1]. In the US, the incidence of PSA is estimated to be approximately 1275 to 3200 new cases per year [2]. Here we report a case of PSA initially presenting with gastrointestinal symptoms. However, it had been misdiagnosed of scleroderma and recurrent chronic gastritis.

## Case presentation

**Fig. 1:**
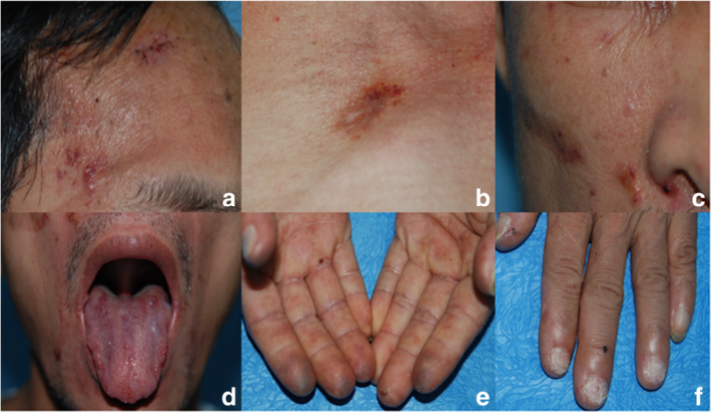
(**a-d**) Petechiae, purpura distributed on the forehead, neck, cheek and tongue; (**e-f**) Digital skin changed as scleroderma

**Fig. 2:**
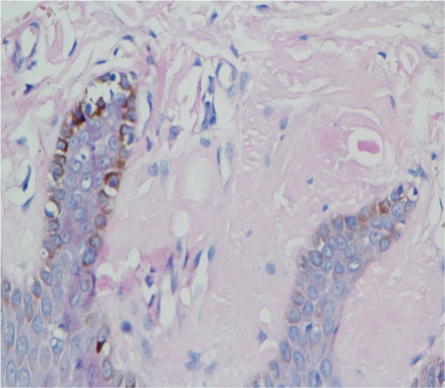
Microphotograph showed pink eosinophilic homogenous material in the dermis, H&E X 40

**Fig. 3:**
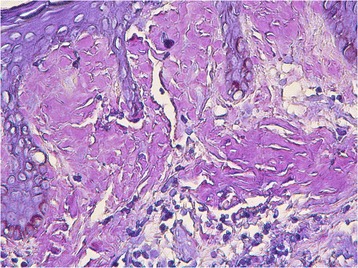
Skin biopsy with crystal violet stain(X 40) showed bright purple amorphous fissured masses in the dermis

**Fig. 4:**
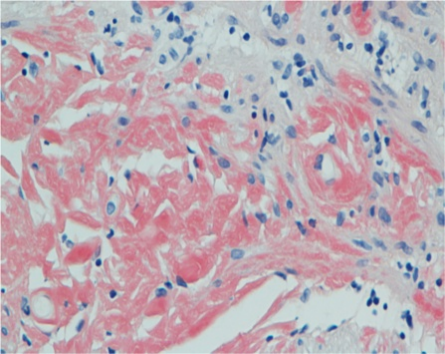
Histopathology of gastric mucosa showed orange-red positivity with Congo Red staining (X 40)

**Fig. 5:**
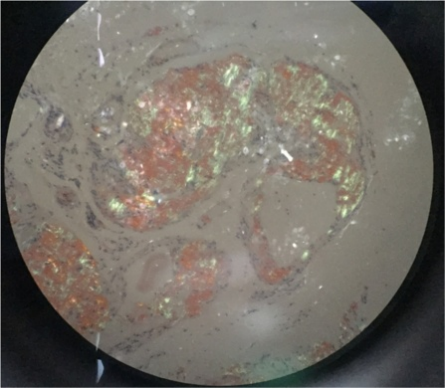
Histopathology of gastric mucosa with Congo Red staining showed characteristic apple-green birefringence under polarized light (X10)

**Fig. 6:**
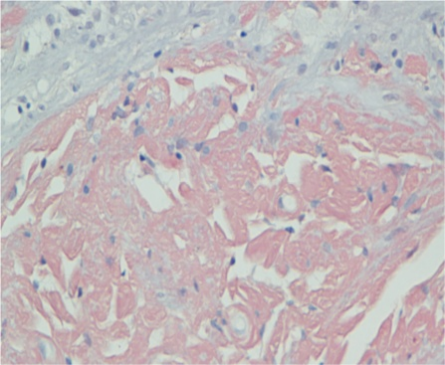
Histopathology of gastric mucosa with Congo Red staining showed pale orange–red after potassium permanganate pretreatment (Microphotograph X40)

A 47-year-old male presented with recurrent dizziness, generalized weakness, easy fatiguablility, accompanied with discontinuous abdominal pain, dyspepsia and constipation for six years. He had been examined with gastroendoscope and magnetic resonance imaging (MRI) of the head for several times, but no specific lesion could be identified. Gradually the skin of his fingers and toes became sclerotic. At the same time, multiple, non-itchy petechiae, purpura and ecchymoses lesions can been found around the eyelids, as well as on the tongue, cheek, neck and the higher chest wall bilaterally (Fig. 1a-f). Biopsy of his palmar skin showed scleroderma-like changes. He had been treated as chronic gastritis and scleroderma. However, his symptoms were aggressive and he came to our hospital. His blood examinations and stool anlysis were found within normal limits. Serum amyloid A protein (SAA) was also negative. Skin biopsy of the forehead lesion showed pink amorphous, eosinophilic fissured masses in the dermis with Haemotoxylin & Eosin staining (Fig. 2). Moreover, it showed purple amorphous fissured masses in dermis with Crystal Violet staining (Fig. 3), which prompted the deposition of amyloid. Besides, histopathologic examination of the gastric mucosal revealed the extensive deposition of amyloid in the gland stomach tissues and vessel wall. The tissue displayed orange–red color with Congo Red staining under the light microscopy (Fig. 4), and showed characteristic apple-green birefringence under polarized light (Fig. 5). Moreover, it showed pale orange–red after potassium permanganate pretreatment (Fig. 6), consistent with the diagnosis of PSA (light chains, AL). Bone marrow biopsy showed a proliferation of 14 % mature plasma cells. Proteinuria electrophoresis showed the presence of Bence-Jones protein. Immunofixation of the concentrated urine detected light chains of LAM-type. Serum and urine immunofixation electrophoresis (IFE) were both positive for monoclonal protein. Analysis of the results showed M proteinemia of IgA-LAM. Quantitative immunoglobulin studies showed a serum IgG value of 3.82 g/l (normal 7–16 g/l), IgA 2.44 g/l (0.7–4 g/l) and IgM 0.20 g/l (0.5–2.20 g/l). Surprisingly, according to the whole abdomen Computed Tomography and ultrasound, we found the patient relied on a solitary right kidney as the left residue was found in the pelvic cavity without any sign of blood flow. Urinalysis showed occult blood and protein quantitative testing for urine protein was 4.1 g/24 h, and blood creatinine and urea nitrogen were in the high level. He was treated with prednisone (40 mg/d), thalidomide, colchicine tablets and gastric mucosa protective drugs. After a month, skin lesion and digestive symptoms improved, but he left hospital because of economic cause.

## Discussion

PSA is a clonal plasma cell disorder, including the production, aggregation, polymerization, fibril formation and finally extracellular deposition in various organs of the precursor protein, ultimately leading to organ dysfunction and death [3]. Symptoms and signs of it are various and complex, which depend on the organs involved. In the early state, patients may present some non-specific clinical symptoms, such fatigue, weight loss, paresthesias and syncopal attacks, just as the patient in this case. Guadually, intracutaneous hemorrhage manifests in the form of petechiae, purpura and ecchymoses due to infiltration of blood vessel walls by amyloid deposits, which are the most common skin lesions. At the same time, symptoms of the affected organs can appear, manifesting multisystem symptoms. In some cases, skin lesions can be atypical, such as multiple papules [4] instead of petechiae and ecchymoses on head and face. In other words, insufficient understanding of the rash is easy to lead to misdiagnosis and delay of treatments.

Firstly, in order to make correct diagnosis of the disease, biopsy with Congo Red staining of the affected organ is significant. Routine pathological examination without the special staining always leads to misdiagnosis [5–7]. As in the case, gastrointestinalscope only presented the feature of chronic erosive gastritis without Congo Red staining. Secondly, distinguishing the type of amyloid is important for treatments. Amyloidosis can be classified clinically into systemic (generalized), with involvement of several organ systems, and organ-limited (localized), in which deposits are limited to a single organ such as the skin (Table 1) [1]. In the systemic amyloidosis, there are primary, secondary and heredofamilial amyloidosis. PSA often accompany with multiple myeloma, which can produce light chain protein as one of the precursor protein of amyloidosis. In this case, treatment of the multiple myeloma is most important to the disease. Thirdly, it is necessary to completely check on the function of important organs, as sedimentation of amyloid can involve any organs. Kidney is the most sensitive organ (74 %) in PSA, then the heart (60 %), liver (27 %) and peripheral nervous system (22 %), autonomic nervous system (18 %) [8]. There is about 69 % of the patients with two organs involvement at least [8]. Gastrointestinal amyloidosis is less common in primary systemic amyloidosis, with only 1 % incidence in a retrospective review from the Mayo Clinic, Rochester, Minnessota, United States [9]. Many endoscopic features of AL amyloidosis are not specific to the disease. Findings include thickened folds, erosions, ulcerations, friability, edema and submucosal hematoma [10–15]. In other words, it is easy to misdiagnosis without biopsy of the lesion with Congo Red staining. The spectrum of renal symptoms and signs in amyloidosis is variable such as isolated proteinuria, nephrotic syndrome, hypertension, hypotension, renal insufficiency [16]. In this case, we did not practice the renal biopsy, as it has been reported that patients of amyloidosis resulted in serious bleeding over renal biopsy centesis [17]. Moreover, examinations revealed that the patient relied on a solitary right kidney as the left one was congenital dysplasia.

**Table 1:** Clinical classification of amyloidosis

Clinical classification of amyloidosis including	
Systemic amyloidosis	
Primary systemic amyloidosis(AL> > AH amyloid protein)	
	Plasma cell dyscrasias (more common)
	Multiple myeloma-associated
Secondary systemic amyloidosis (reactive;AA amyloid protein)	
	Chronic inflammation (e.g.rheumatoid arthritis)
	Chronic infection (e.g.tuberculosis)
Heredofamilial amyloidosis (various amyloid proteins)	
	Familial amyloidotic polyneuropathy
	Familial Mediterranean fever
Organ-limited (localized)amyloidosis	
Cutaneous	
	Primary:macular, lichen, biphasic, dyschromic, nodular
	Secondary:within skin tumors
Endocrine	
	Medullary carcinoma of the thyroid, insulinoma, type 2 diabetes
Cerebral	
	Alzheimer 's disease

## Conclusions

In summary, clinical manifestations of PSA are diverse and nonspecific. It should be considered in patients with skin lesion such as petechiae, purpura and ecchymoses, especially accompanying with multisystemic symptoms. Biopsy of lesion with Congo Red staining is of diagnostic significance.

## Consent

Written informed consent was obtained from the patient for publication of this Case Report and any accompanying images.
